# Identification and functional characterisation of a novel DNASE1L3 variant (c.572A>G, p.Asn191Ser) in three Emirati families with systemic lupus erythematosus and hypocomplementaemic urticarial vasculitis

**DOI:** 10.1136/lupus-2024-001477

**Published:** 2025-02-13

**Authors:** Najla Aljaberi, Anjali Bharathan, Remya Prajesh Gopal, Ekhlass Mohammed, Fatema Al Shibli, Mohammed Tabouni, Sara Alhmoudi, Praseetha Kizhakkedath, Ibrahim Baydoun, Mushal Allam, Noor Mustafa, Fatma Aljasmi, Afra Al Dhaheri, Hiba Alblooshi

**Affiliations:** 1 Department of Pediatrics, United Arab Emirates University College of Medicine and Health Sciences, Al Ain, Abu Dhabi, UAE; 2 Department of Genetics and Genomics, United Arab Emirates University College of Medicine and Health Sciences, Al Ain, Abu Dhabi, UAE; 3 Rheumatology Department, Tawam Hospital, Al Ain, Abu Dhabi, UAE

**Keywords:** Systemic Lupus Erythematosus, Polymorphism, Genetic, Vasculitis

## Abstract

**Objectives:**

To evaluate the functional impact of a novel *DNASE1L3* variant (c.572A>G, p.Asn191Ser) in three families with SLE and hypocomplementaemic urticarial vasculitis (HUV) from the United Arab Emirates.

**Methods:**

Whole-exome sequencing was performed on affected patients and findings were confirmed using Sanger sequencing in family members. DNASE1L3 protein expression, secretion and enzymatic activity were assessed in HEK293 cell lines. Plasma smear assay for neutrophil extracellular traps (NETs) was evaluated in patients, family members and healthy control.

**Results:**

A total of seven patients diagnosed with both SLE and HUV were identified from three unrelated families. All affected individuals were found to carry a homozygous c.572A>G, p.Asn191Ser (191S) variant in *DNASE1L3*. The variant 191S was shown to impact the secretion and activity of DNASE1L3. Patients homozygous for 191S variant had significantly higher burden (p=0.0409) of NET structure in comparison to heterozygous and healthy control.

**Conclusions:**

We functionally evaluated the effect of a novel *DNASE1L3* (c.572A>G, p.Asn191Ser) in familial SLE with a consistent pattern of HUV across seven patients. This variant resulted in impaired secretion and enzymatic activity of DNASE1L3 along with increased NETosis in patients with homozygous genotype.

**WHAT IS ALREADY KNOWN ON THIS TOPIC**

- *DNASE1L3* variants are linked to familial SLE and hypocomplementaemic urticarial vasculitis (HUV). The majority of reported variants are loss-of-function variants leading to abolition of enzyme activity and development of autoimmunity.

**WHAT THIS STUDY ADDS**

- The novel variant *DNASE1L3* (c.572A>G, p.Asn191Ser), when inherited in homozygous form, leads to SLE and HUV clinical manifestations.
- *DNASE1L3* (c.572A>G, p.Asn191Ser) leads to aberrant enzyme secretion and catalytic activity.
- Patients with homozygous mutations in *DNASE1L3* (c.572A>G, p.Asn191Ser) have a significantly higher level of peripheral neutrophil extracellular trap formation compared with heterozygous carriers and healthy control.

**HOW THIS STUDY MIGHT AFFECT RESEARCH, PRACTICE OR POLICY**

- This work delineates a functional defect in DNASE1L3 secretion and activity which emphasises its important role as a potential therapeutic target in SLE.

## Background

 Deoxyribonuclease 1-like 3 (DNASE1L3) is an endonuclease that belongs to the DNASE I family and is encoded by the *DNASE1L3* gene. It is primarily secreted by macrophages and dendritic cells and is expressed mainly in the liver and lymphoid organs.^1^ Compared with DNASE 1, DNASE1L3 has the ability to cleave both ‘naked’ DNA and DNA in microparticles of lipids and proteins.^2 3^ Such an essential function of digesting chromatin microparticles produced by apoptotic cells is important in the elimination of antigenic DNA and autoreactivity.^4^,^6^ The loss of such function has been linked to the development of SLE in both animal models and humans.^7^,^16^ A loss-of-function variant in the *DNASE1L3* gene in patients was first reported by Al-Mayouf *et al* in seven Arab families.^9^ Several reports outlining genetic variants were identified since then, further linking DNASE1L3 to the development of SLE as well as the distinct hypocomplementaemic urticarial vasculitis (HUV) rash.^10^,^1214 17^ Patients with SLE often have various forms of cutaneous manifestations including HUV which occurs in up to 5–10% of patients with SLE.^24 25^ However, there is a recurrent pattern of this form of rash in many patients with pathological variants in *DNASE1L3.*^10^,^1223^ The emergence of increasing numbers of clinical reports linking *DNASE1L3* mutations to SLE highlights the key role of this enzyme in the pathogenesis of SLE. On a larger scale, genome-wide association studies have demonstrated the association of missense single nucleotide polymorphism in *DNASE1L3* with SLE, scleroderma and rheumatoid arthritis.^13 26^ In this report, we demonstrate the identification and functional assessment of a novel *DNASE1L3* variant (c.572A>G, p.Asn191Ser) causing SLE and HUV in three unrelated families from the United Arab Emirates (UAE).

## Methods

### Patients and ethical approval

Patients with SLE (n=7), their unaffected relatives (n=8) and one healthy control were recruited as part of an ongoing SLE genetic registry project (IRB number: DOH/CVDC/2023/486) at UAE University Genomics Laboratory. Informed consent was obtained for all participants. Patient photographs were taken with additional written consent for publication. Blood samples were collected for laboratory processing. Other disease metric data were retrieved, including clinical and laboratory data from the patients’ electronic health records.

### Sample processing

Genomic DNA (gDNA) was extracted using the QIAcube automated DNA extraction system with the QIAamp DNA Mini Kit (QIAGEN) from peripheral blood samples collected in EDTA tubes. The gDNA quality was assessed using spectrophotometric (NanoDrop, Thermo Fisher Scientific, Waltham, Massachusetts, USA), and the DNA quantity was evaluated via fluorometric quantitation method (Qubit 4.0 fluorometer, Thermo Fisher Scientific). DNA fragmentation was performed using a Covaris LE-220 plus ultrasonication system (Covaris, Woburn, Massachusetts, USA). Whole-exome sequencing (WES) library preparation and Exome capture were performed by the TruSeq DNA Exome (Illumina, San Diego, California, USA.) following the manufacturer’s protocol. Sequencing was performed using S2 flow cell with paired-end reads (2×150 bp) on NovaSeq 6000 system (Illumina). A combination of in-house-developed pipelines and the Illumina DRAGEN Bio-IT Platform (Illumina) was used for read mapping, alignment and variant calling. VarSeq V.2.2.4 software (Golden Helix, USA) was used for variant annotation and filtration.

The identified variants were clinically correlated with relevant inheritance patterns based on the information provided by the referring physician regarding clinical data and family history. Subsequently, the filtered variants were interpreted using the guidelines established by the American College of Medical Genetics and Genomics, while also considering the patient’s phenotype.

### Segregation analysis of *DNASE1L3* (c.572A>G, p.Asp191Ser) variant

*DNASE1L3* (c.572A>G, p.Asp191Ser) variant was detected in probands from families A, B and C via WES. PCR was carried out using PCR Master Mix Kit (QIAGEN, Germany) to amplify the region of interest using the designed primers.

Forward primer: 5′GCACCCACCAGGTGACAGT 3′

Reverse primer: 5′ACCAAGCACTGTGGTGAGC 3′

The finding was validated using ABI 3130xl genetic analyser (Applied Biosystems, Waltham, Massachusetts, USA) according to the manufacturer’s protocol.

### In silico prediction of DNASE1L3 protein

Predict Protein (https://predictprotein.org)^27^ was used to evaluate the functional effect of the *DNASE1L3* missense variant c.572A>G or p.Asp191Ser for protein sequence analysis. An in silico tool SWISS MODEL^28^ was used to generate homology models of wild-type (191N) and mutant (191S) human protein structures. PyMOL was used to evaluate and visualise the generated models.^29^

### Plasmid construct and site-directed mutagenesis

DNASE gamma (DNASE1L3) (NM_004944) Myc-DDK-tagged Human Tagged ORF Clone (OriGene, USA; Cat No RC205611) was used to introduce c.572A>G, p.Asp191Ser into the clone by site-directed mutagenesis (SDM) using PfuUltra HF polymerase (Agilent, USA). The primers for introducing the variant are listed below:

Forward primer: 5′CATTTTCATGGGTGACTTCAGTGCCGGCTGCAGCTACGTC 3′

Reverse primer: 5′GACGTAGCTGCAGCCGGCACTGAAGTCACCCATGAAAATG 3′

The SDM of the clones was confirmed using ABI 3130xl genetic analyser (Applied Biosystems).

### Cell line culture, transfection and preparation

Human embryonic kidney cells (HEK293; ATCC, USA) were cultured in Dulbecco’s Modified Eagle Medium (Invitrogen) supplemented with 10% fetal bovine serum (Gibco, 10500-064) and penicillin (10 U/mL)/streptomycin (100 μg/mL) (Gibco; Cat No 15140-122) at 37°C in 5% CO_2_. Cells were grown in six-well tissue culture plates and transfected with 1 μg of plasmid DNA using FuGENEÒHD (Promega, USA; Cat No E2311) transfection reagent according to manufacturer’s protocol. To assess secretion blockage, cells were treated with 1 µL BD brefeldin A (BFA) GolgiPlugÔ Protein Transport inhibitor (BD, USA; Cat No 555029) according to the manufacturer’s protocol.

### Protein extraction

48 hours after transfection, total protein was extracted using 1X RIPA lysis buffer (Thermo Fisher Scientific; Cat No 89901) containing Halt protease and phosphatase inhibitor cocktail (Thermo Fisher Scientific; Cat No 78441).

The cell supernatant was collected at 24, 48 and 72 hours after transfection. Then the supernatants were filtered through 0.22 µm centrifuge tube filters (Costar Spin-X; Cat No 8160). The total protein lysate and supernatant were quantified using Pierce BCA Protein Assay Kit (Thermo Fisher Scientific; Cat No 23225). Optical density was measured at 490 nm using the TECAN Infinite M200 Pro plate reader (TECAN, Switzerland).

### Western blot

The lysates and supernatants were loaded on 10% sodium dodecyl sulfate-polyacrylamide gel electrophoresis. After electrophoresis, the gels were immunoblotted onto polyvinylidene fluoride (PVDF) membranes (Thermo Fisher Scientific; Cat No 88518). The membranes were blocked in 5% bovine serum albumin (BSA) (Sigma; Cat No A2153) for 1 hour at room temperature and incubated overnight in the primary antibody at 4°C. The membranes were washed four times in phosphate-buffered saline with Tween 20 (PBST) and incubated in horseradish peroxidase (HRP)-conjugated secondary antibody for 1 hour at room temperature. The bands were detected by PierceäECL Western Blotting Substrate as per the manufacturer’s instructions (Thermo Fisher Scientific; Cat No 32106) in a Sapphire Biomolecular Imager, Azure Biosystems (Azure Biosystem, USA). The antibodies used and their dilutions were as follows: DYKDDDDK tag (D6W5B) rabbit mAb (1:1000, Cell Signaling Tech, Massachusetts; Cat No 14793), rabbit mAb β-Actin (1:1000, Cell Signaling Tech; Cat No 4970) and mouse anti-rabbit immunoglobulin G (IgG)-HRP (1:5000, Santa Cruz; Cat No sc-2357).

### ELISA

DNASE1L3 protein was quantified from cell lysates and supernatants using Human Deoxyribonuclease Gamma (DNASE1L3) ELISA Kit (MyBioSource; Cat No MBS925159) as per the manufacturer’s protocol. A total of 100 µL of cell lysates and supernatants before and after treatment with BFA were added into the wells of precoated microplate. The colour intensity was determined by TECAN Infinite M200 Pro plate reader at 450 nm. Experiments were repeated at least three times and the mean±SE calculated.

### Plasmid-nicking assay

To examine the endonuclease activity of DNASE1L3, a plasmid-nicking assay was performed using a supercoiled pBR322 plasmid as a substrate (New England Biolabs, USA), following Al-Mayouf *et al*.^9^ Supernatants of 191N and 191S (undiluted and 1:10 diluted) were added to pBR322 plasmid (final concentration 25 µg/mL) dissolved in 25 mM HEPES, 1 mM MgCl_2_, 1 mM CaCl_2_, 100 μg/mL BSA to a total reaction volume of 30 mL. The reaction mixture was incubated for 30 min at room temperature. A linearised pBR322 with EcoRI digestion was used as a positive control (New England Biolabs, USA). Enzymatic reactions were stopped by adding EDTA (final concentration: 10 mM/30 µL). Comparison was done between 191N and 191S DNASE1L3 proteins observed via agarose gel electrophoresis and visualisation of ethidium bromide-stained plasmid DNA bands under ultraviolet light.

### Plasmid DNase enzymatic activity assay using TaqMan quantitative PCR

Endonuclease activity of DNASE1L3 was measured using Plasmid DNase enzyme activity assay following Coke *et al*.^13^ GFP plasmid Pcdna3.1+N-eGFP (Addgene, USA) was used as a DNase substrate. Plasmid was prepared by QIAprep Miniprep Kit (QIAGEN, Germany) following manufacturer’s protocol. A total of 45 μL of supernatants and cell lysates were combined with 5 μL of substrate plasmid (1 ng/μL) and incubated at 37°C overnight. Then the reaction was stopped by cooling to 4°C. Reaction mixtures were diluted 100 times before TaqMan quantitative PCR (qPCR) assay. Commercial DNase I (Thermo Fisher Scientific) was spiked into supernatants and lysates of untransfected cells used as positive control. A qPCR assay was performed to quantify the plasmid DNA using TaqMan Assay probe for GFP (Mr00660654_cn).

### Plasma neutrophil extracellular trap smear assay

Plasma was separated from patients’ whole blood by centrifugation at 3000 RPM for 10 min at 4°C. A total of 1 μL of patient plasma was smeared on poly-L-lysine (Sigma-Aldrich, USA) coated glass slides following the protocol in Matta *et al*.^30^ The samples were fixed with 4% formaldehyde for 10 min. The slides were washed three times with phosphate-buffered saline (PBS). Fixed slides were then blocked with 5% BSA/PBS for 1 hour and then stained with anti-MPO (Abcam number: 25989) and anti-CitH3 (Abcam number: 5103) antibodies (diluted to 1 µg/mL in 0.3% Triton X-100+0.1% BSA) overnight in moist chamber. On the next day, slides were washed three times with PBS and then incubated with goat anti-mouse Alexa Fluor 488 (IgG (H+L), 2 mg/mL, Invitrogen A11029) and goat anti-rabbit Alexa Fluor 594 (IgG (H+L), 1 mg/mL, Invitrogen 35560) secondary antibodies (diluted 1:500) for 1 hour. After washing with PBS, slides were stained with 5 mg/mL DAPI (Sigma-Aldrich, D9542) for 15 min. After final three washes, the cover slip was mounted in Vecta Mount AQ Mounting Medium (VectorLabs number: H-5501). Images were taken at 20× using Leica MICA Microhub fluorescence microscope system (Leica Microsystems, Germany). The area with the highest intensity was captured for further analysis. The captured neutrophil extracellular trap (NET) images were then analysed using ImageJ Java-based software (V.1.8) measuring average intensity of the threshold area, representing circulating NETs. The images were first converted into 8-bit greyscale images. Threshold limits were set to pixel intensity range of 40 min to 255 max for each image.

### Statistical analysis

Data were analysed using GraphPad Prism V.10.0.0 (GraphPad, San Diego, California). Differences between two groups were determined with an unpaired two-tailed Student’s t-test. For three groups, one-way analysis of variance tests were performed. Significance was established at p<0.05. For the quantification of the NET a two-tailed parametric test was performed for the comparisons between samples with normal distribution. Quantification of DNASE1L3 enzymatic activity was analysed by comparative Ct (∆∆Ct) method.

## Results

### Identification and clinical description of affected patients with *DNASE1L3* (c.572A>G, p.Asn191Ser)

**Figure 1:**
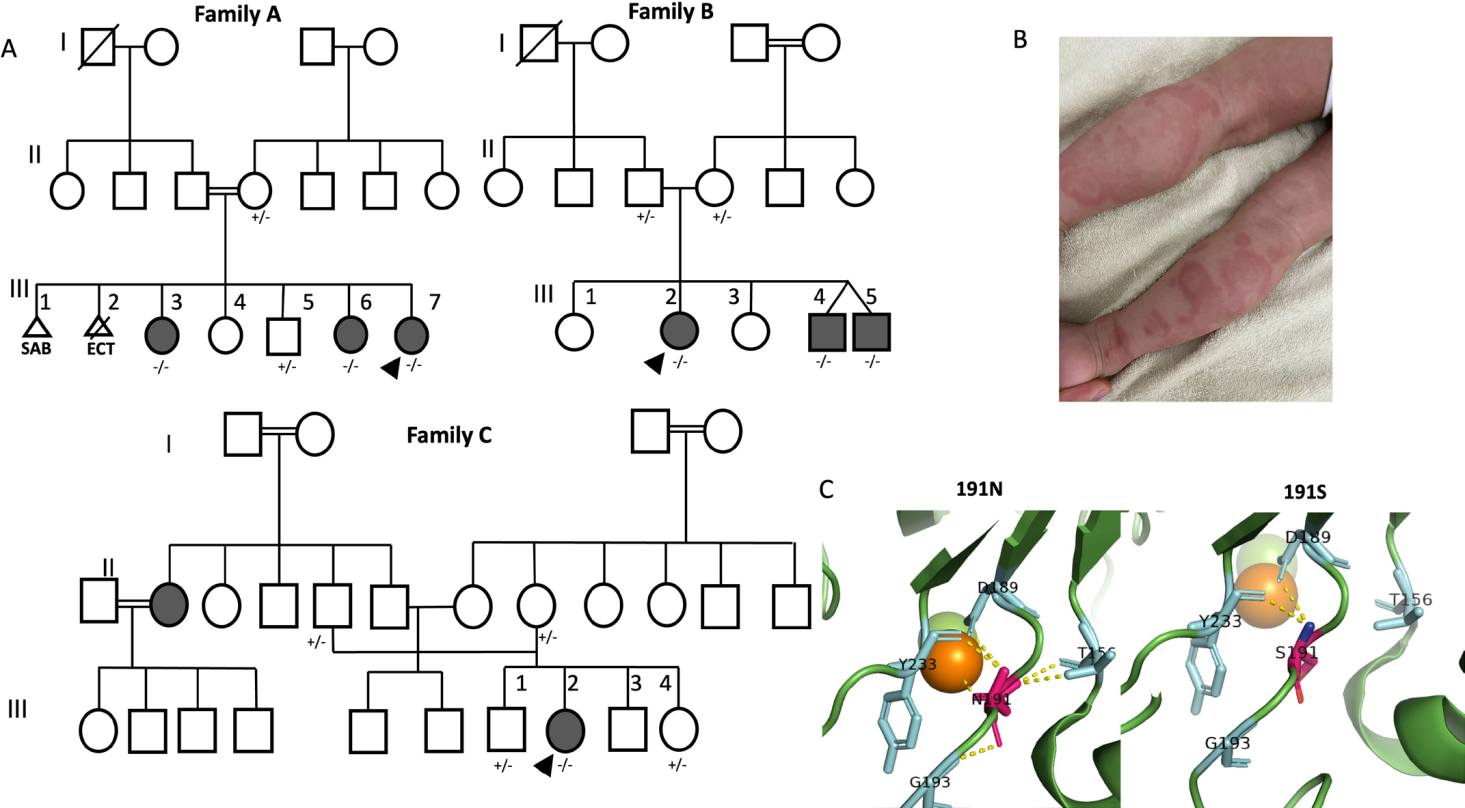
(**A**) Pedigrees of the studied families (family A, family B and family C) and segregation of *DNASE1L3* c.572A>G, p.Asn191Ser in tested family members where +/+ denotes homozygous wild type, ± heterozygous and −/− homozygous mutant. (**B**) Skin lesions of urticarial vasculitis (biopsy image not available) of patient III.7 from family A. (**C**) Generated homology model of missense variant in *DNASE1L3* (c.572A>G, p.Asn191Ser), the amino acid is represented as sticks, Mg^+2^ represented as spheres. The left 191N is asparagine residue at the 191st position in the wild type. The right 191S is serine residue at 191st position in the mutant which highlights the disrupted interaction with Mg^+2^.

Identified patients with familial SLE were enrolled in this study. All seven subjects from three unrelated families (from the same ancestral tribe) had a certain extent of a clinical phenotype ranging from early SLE findings to fully classified SLE.^31^ The pedigree of patients and family members illustrates the segregation of *DNASE1L3* (c.572A>G, p.Asn191Ser) variant (figure 1A). The protein prediction model demonstrated disrupted interaction with Mg^+2^ molecule as shown in figure 1C. This variant has not been observed in large population databases such as Genome Aggregation Database prior to our initial reporting of the variant^32^ (ClinVar ID: 3248531). The clinical description of all patients is presented in table 1. Age of onset is defined as the earliest point the patients presented with either SLE or HUV. All subjects demonstrated HUV clinically (figure 1B) and three had consistent histopathological findings on skin biopsy.

**Table 1:** Clinical and laboratory features of patients with *DNASE1L3* (c.572 A>G, p.Asn191Ser) mutation

Families	Family A			Family B			Family C
Patients	III.3	III.6	III.7	III.2	III.4	III.5	III.2
Age at onset	3 years	2 years	13 months	2 years	3 years	3 years	13 years
Age at SLE diagnosis^31^ (years)	3	6	–	3	–	–	13
Current age (years)	19	9	4	9	4	4	13
**Clinical findings**							
HUV	+*	+	+*	+*	+	+	+
Fever	+	+	+	–	–	–	+
Lymphadenopathy	–	+	+	–	–	–	+
Lupus nephritis	Class IV	Class II/V	–	–	–	–	Class IV/V
**Laboratory findings**							
Positive ANA	+	+	–	+	+	+	+
Positive anti-dsDNA	+	+	+	+	+	–	+
Positive ANCA	–	–	N/A	–	N/A	N/A	N/A
Low C3/low C4	+/+	+/−	+/−	+/+	+/−	+/+	+/+
Low C1q	N/A	–	+	+	+	+	N/A
Positive aPLs	–	–	–	–	N/A	N/A	–
**↓**Hgb/**↓**WBC/**↓**plt	+/−/−	−/+/−	+/−/−	+/−/−	+/−/−	−/−/−	+/+/−
Immunomodulator history	HCQ, MMF, belimumab, rituximab	HCQ	HCQ	HCQ	–	–	HCQ, MMF, rituximab,CYC
Outcomes	Intermittent proteinuria and HUV (mild). Normal renal function	In remission (systemic and renal). Normal renal function	Persistent HUV despite HCQ	Intermittent HUV (mild)	Intermittent HUV (mild)	Intermittent HUV (mild)	CKD despite induction with steroids, rituximab, MMF, CYC and plasma exchange

* Confirmed with skin biopsy.

ANCA, anti-neutrophil cytoplasmic antibodies; aPLantiphospholipid antibodiesCKD, chronic kidney diseaseCYC, cyclophosphamide; HCQ, hydroxychloroquine; Hgb, haemoglobin; HUV, hypocomplementaemic urticarial vasculitis; MMF, mycophenolate mofetil; N/A, not available; plt, plateletsWBCwhite blood cell

**Figure 2:**
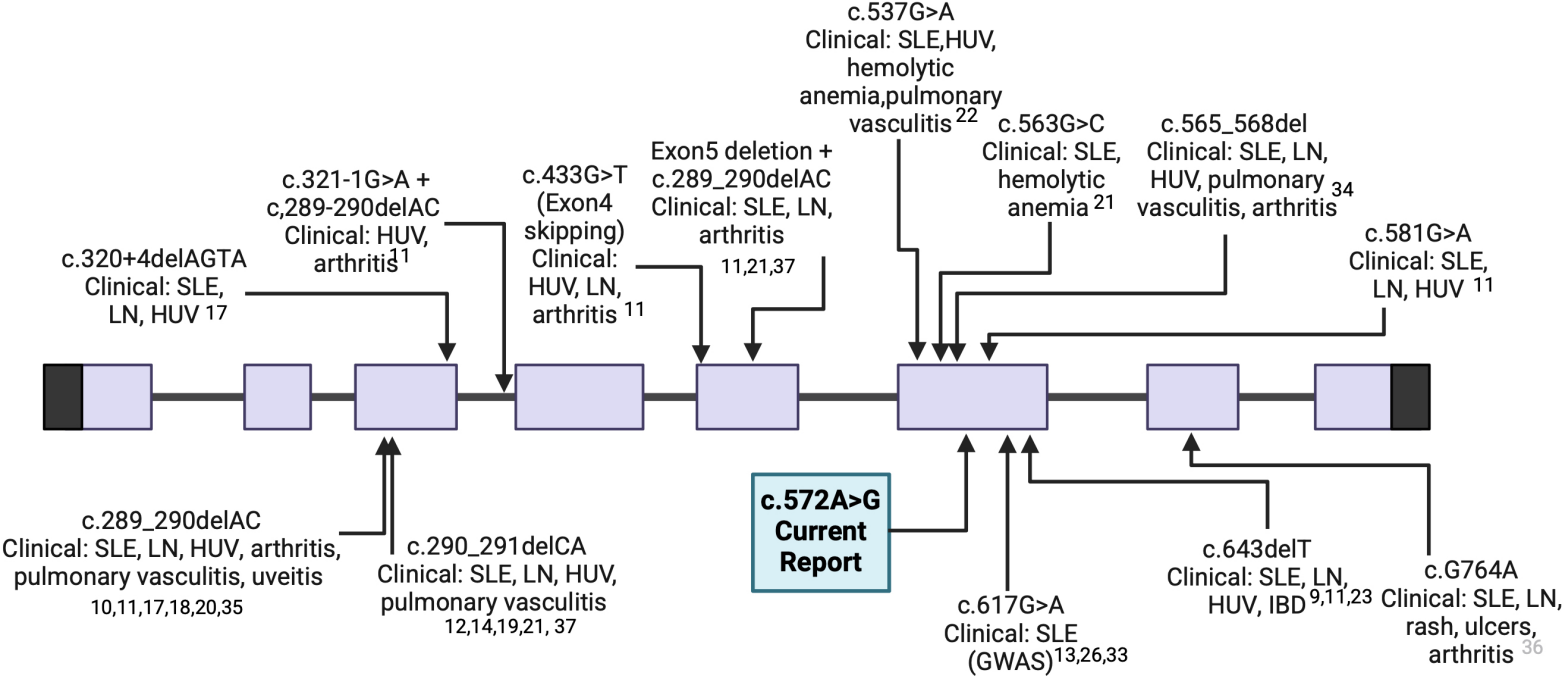
Summary of reported *DNASE1L3* variants aligned with the exonic location and the patients’ corresponding clinical presentation. Our variant is indicated by the box at exon 6 (image generated by BioRender). GWAS, genome-wide association studies; HUV, hypocomplementaemic urticarial vasculitis; IBD, inflammatory bowel disease; LN, lupus nephritis.

Figure 2 outlines our variant in comparison with other reported variants.^9^,^1417^ The clinical phenotype in our patients overlaps with that of reported patients highlighting HUV as a recurrent feature along with SLE. Exon 6 is considered to be a hot spot for many of the previously reported variants.

### Homozygous 191S variant of *DNASE1L3* leads to overproduction of intracellular DNASE1L3 enzyme but marked reduction in the secreted form

**Figure 3:**
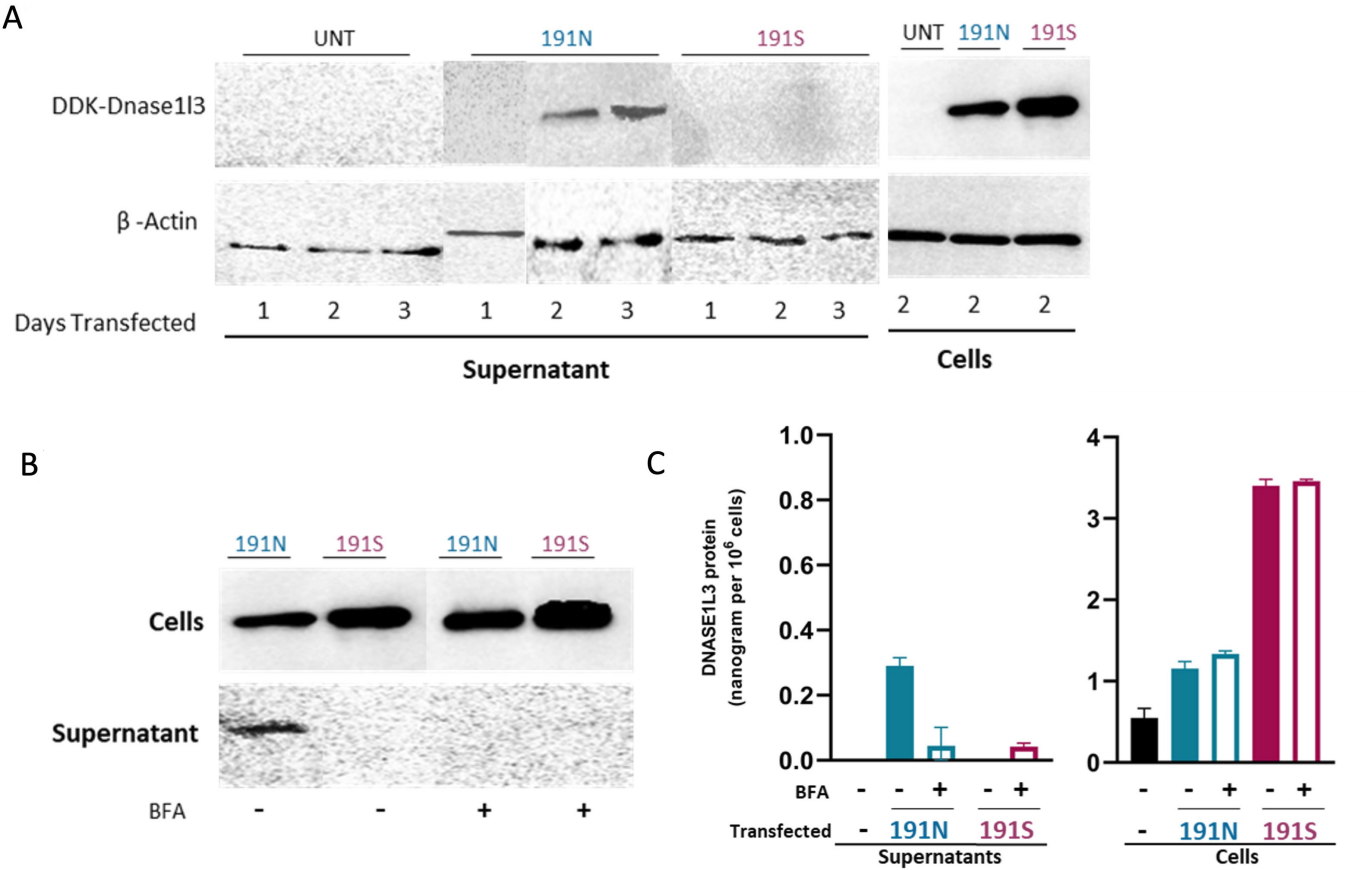
*DNASE1L3* c.572A>G, p.Asn191Ser variant (191S) does not affect enzyme production, but it affects the secretion of the enzyme. (**A**) In transfected human embryonic kidney (HEK293) cells, DNASE1L3 191N protein was produced normally while 191S protein was overexpressed. In the supernatant, DNASE1L3 191N protein was detected but not 191S protein. (**B**) Secretion of DNASE1L3 191N protein was normally blocked by Golgi blocker brefeldin A (BFA), while there was no secretion of DNASE1L3 191S protein in either condition. (**C**) DNASE1L3 191N and 191S protein levels measured by ELISA in cells and supernatants with and without BFA, reflecting the levels measured on western blot.

Transfected HEK293 cells with 191S variant construct showed overexpression of DNASE1L3 protein intracellularly when compared with wild-type 191N cells (figure 3A). The difference in the intracellular amount of detected DNASE1L3 was observed visually and quantitatively (figure 2). As expected, treatment with BFA blocked the secretion of DNASE1L3 in both 191N and 191S supernatants and led to the accumulation of intracellular DNASE1L3 in both cell lines. The amount of accumulated DNASE1L3 is again shown to be significantly higher in 191S cell lines (figure 3B,C). As demonstrated, the secretion of DNASE1L3 191S protein is consistently deficient compared with 191N. This signifies the detrimental effect of this variant on the secretion of DNASE1L3 enzyme which mainly acts extracellularly.

### Homozygous 191S variant of *DNASE1L3* results in abnormal reduction of the catalytic function of DNASE1L3 enzyme

**Figure 4:**
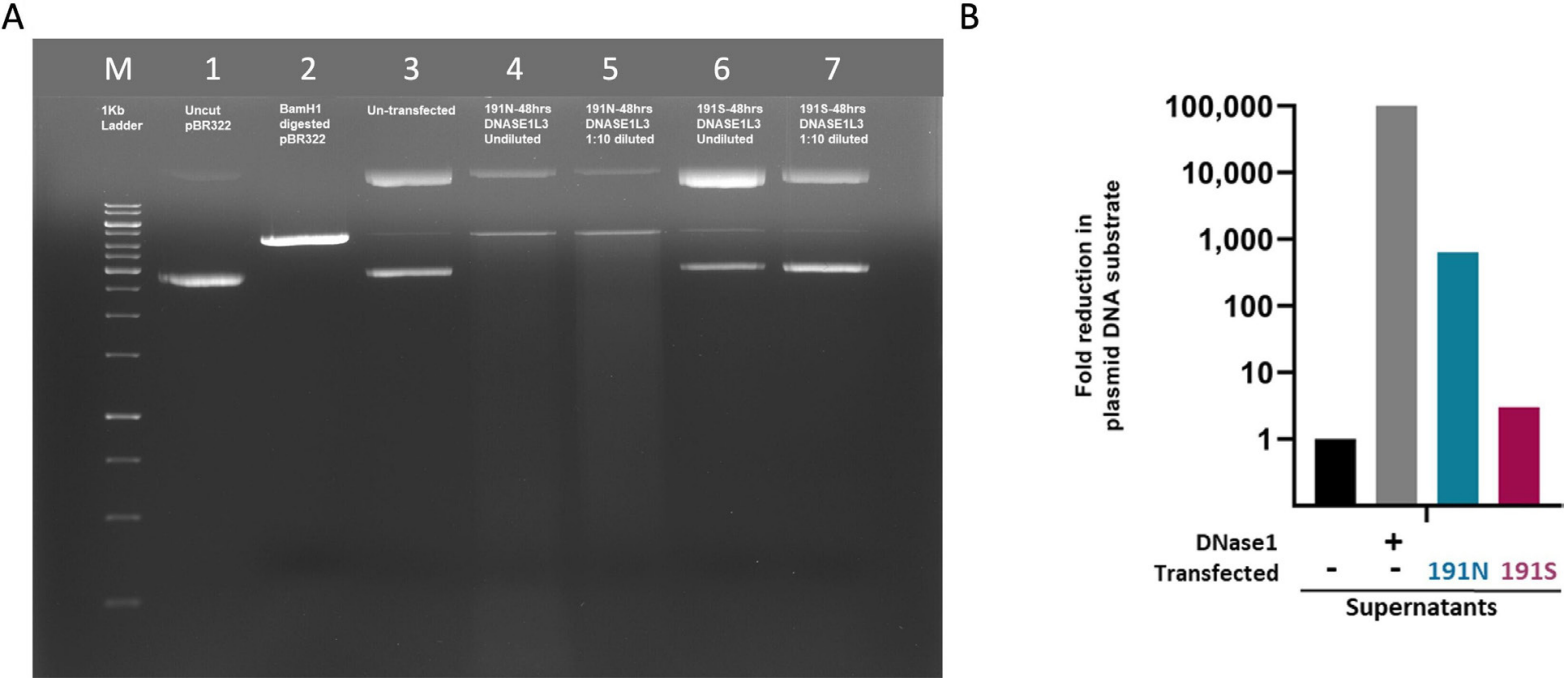
*DNASE1L3* c.572A>G, p.Asn191Ser variant (191S) demonstrates abnormally reduced function on plasmid-nicking assay. (**A**) Plasmid-nicking assay demonstrated on agarose gel electrophoresis using pBR322 plasmid as a substrate. M: 1 kb DNA size ladder. Lane 1: uncut pRB322 plasmid. Lane 2: digested pRB322 plasmid. Lane 3: untransfected cells. Lane 4: undiluted medium from transfected cells with 191N. Lane 5: 1:10 dilution of medium from cells transfected with 191N. Lane 6: undiluted medium from cells transfected with 191S. Lane 7: 1:10 dilution of medium from cells transfected with 191S. (**B**) Quantification of the endonuclease plasmid-nicking activity of DNASE1L3. A quantitative PCR (qPCR) assay was performed to quantify the plasmid DNA using TaqMan probe for GFP. Fold reduction in plasmid DNA substrate was measured and compared with reference level in untransfected cells. The level of plasmid DNA nicking was reduced in DNASE1L3 191S compared with DNASE1 and DNASE1L3 191N.

Using pBR322 plasmid as a substrate, we assessed the catalytic activity of 191N and 191S DNASE1L3 protein. Fragmentation of the incubated plasmid substrate was visibly reduced in 191S when looking at the agarose gel electrophoresis bands (figure 4A). The DNase activity was assessed quantitatively by measuring the fold reduction of the plasmid substrate and comparing it to the reference level in untransfected cells. This has shown considerable and near-complete lack of DNase activity in 191S variant (figure 4B).

### Circulating NETs are present in higher amounts in patients with homozygous 191S variant of *DNASE1L3* compared with heterozygous carriers and healthy controls

**Figure 5:**
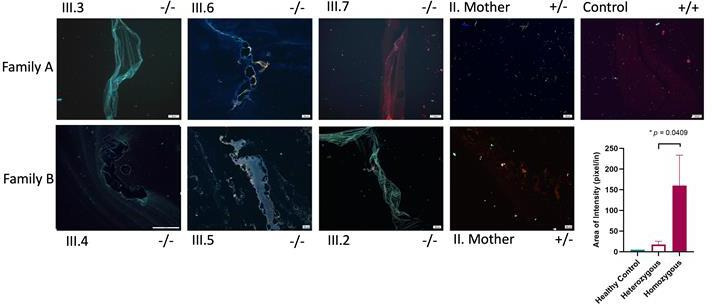
Plasma smear assay showing higher neutrophil extracellular traps (NETs) in patients with SLE with *DNASE1L3* c.572A>G, p.Asn191Ser variant (191S) from family A (III.3, III.6, III.7) and family B (III.4, III.5, III.2) compared with heterozygous carriers of 191S (II. Mother) from both families and healthy control. Representative merged images of plasma smeared poly-L-lysin slides stained with MPO (green, AF488), CitH3 (red, AF594) and DAPI (blue). All images were taken on MICA imaging system (Leica) at 20× objective. Set scale at 100 µm. Plasma smear quantification using average threshold-area pixel intensity using ImageJ (bottom right) showing significantly higher area of intensity for NETs in 191S patients compared with heterozygous carriers of 191S and healthy control.

The levels of circulating NETs in plasma samples of six of the patients, their mothers and a healthy control were assessed visually. Using plasma smear assay, NET structures were clearly formed in patients with homozygous 191S in comparison to heterozygous carriers and healthy control (figure 5). The plasma smeared poly-L-lysine single-channel images of the individual stains are shown in online supplemental figure 1. The level of pixel intensity on the captured images showed a significantly higher level of area intensity indicating higher levels of plasma NETs in homozygous 191S (figure 5). Further assessment of the correlation between NET formation and disease severity via SLE Disease Activity Index scores was conducted in affected patients. This did not reveal a significant correlation, although it was a small sample size (data not shown).

## Discussion

DNASE1L3 has emerged as an instrumental player in the formation of autoimmunity linked with SLE. We outline a consistent pattern of early-onset SLE in our affected patients along with biopsy evidence of HUV, in line with several reports in the literature.^10^,^1223^ Most of what has been reported in the literature regarding *DNASE1L3* variants were loss-of-function variants.^9^,^1214 17^ Here we report the effect of a novel missense variant (c.572A>G, p.Asn191Ser) in *DNASE1L3* on phenotypic SLE with HUV. The variant was found in three unrelated families from the same ancestral tribe indicating potential founder effect.^38^ Given that DNASE1L3 is a Ca^+2^/Mg^+2^-dependent nuclease,^1^ 191S variant’s disruption of the Mg-binding site in our homology model may explain the functional influence on the enzyme’s secretion and/or catalytic activity.

Both DNASE1 and DNASE1L3 play a role as serum nucleases.^3^ However, DNASE1L3 has been found to have broader substrates such as DNA complexed in microparticles of proteins and lipids.^5 39^ Despite the complementary functions of DNASE1 and DNASE1L3, the deficiency of DNASE1L3 results in overt autoimmunity that is not compensated for by DNASE1.^2^ This vital role in digesting circulating microparticles explains the importance of the extracellular function of secreted DNASE1L3 in eliminating autoantigens.^6^ In our study, we demonstrated that 191S variant primarily affected the secretion of DNASE1L3 in the extracellular space and led to retention of enzyme intracellularly. The consequence of this aberrant secretion likely leads to a higher burden of circulating autoantigens that would otherwise be processed by DNASE1L3. The catalytic activity of the DNASE1L3 was evaluated and showed the 191S variant led to marked reduction but not complete abolishment of the enzymatic activity. Both observations align with prior evidence of other missense variants (Arg 206Cys) resulting in aberrant secretion and relative reduction in DNASE1L3 function.^13^

NETosis as a source of highly autoantigenic material is considered a prominent component in the pathogenesis of SLE.^40^ Serum DNases, including DNASE1 and DNAS1L3, have been found to play a role in processing NET structure, thereby promoting self-tolerance.^39 41^ In our study, we demonstrated the high burden of NETs in patients with aberrant DNASE1L3 function (191S variant) in comparison to heterozygous carriers and healthy control. Several studies showed the correlation between NETosis and SLE disease activity.^42 43^ In our patients with variable SLE disease activity, including those who do not yet fulfil SLE criteria, all had considerable burden of NETosis. This subclinical generation of NETs might be an intrinsic aspect of the abnormal activity of DNASE1L3, preceding the onset of a fully manifested SLE phenotype.

The findings in our study highlight the vital role of DNASE1L3 as a housekeeping endonuclease in a cell-extrinsic fashion signifying the importance of normal enzyme secretion. Deficiency in this function was reflected in the formation of NETs which significantly correlated with the homozygous carrier state. Investigating missense variants at the functional level underscores the significance of comprehending the pathogenesis of SLE, particularly in familial cases.

Our findings would be more robust with the replication of the enzyme assay using additional patient samples, which are currently unavailable. Despite the limitation of having only one healthy control sample for the plasma smear assay, our results demonstrated a clear distinction between patient samples and those from unaffected individuals (heterozygous carriers and the healthy control).

## supplementary material

10.1136/lupus-2024-001477 online supplemental file 1

## Data Availability

Data are available upon reasonable request.
