# Supplementary material for: Identification and functional characterisation of a novel DNASE1L3 variant (c.572A>G, p.Asn191Ser) in three Emirati families with systemic lupus erythematosus and hypocomplementaemic urticarial vasculitis
Source: Lupus Sci Med. 2025 Feb 13;12(1):e001477. doi: 10.1136/lupus-2024-001477 (PMC11831315; doi:10.1136/lupus-2024-001477)

Supplemntary Figure 1: The plasma smeared poly-l-lysine single channel images of the individual stains MPO (green, AF488), CitH3 (red, AF594), and DAPI (blue).

Family A

Merged **Cit-H3** **MPO** **DAPI**

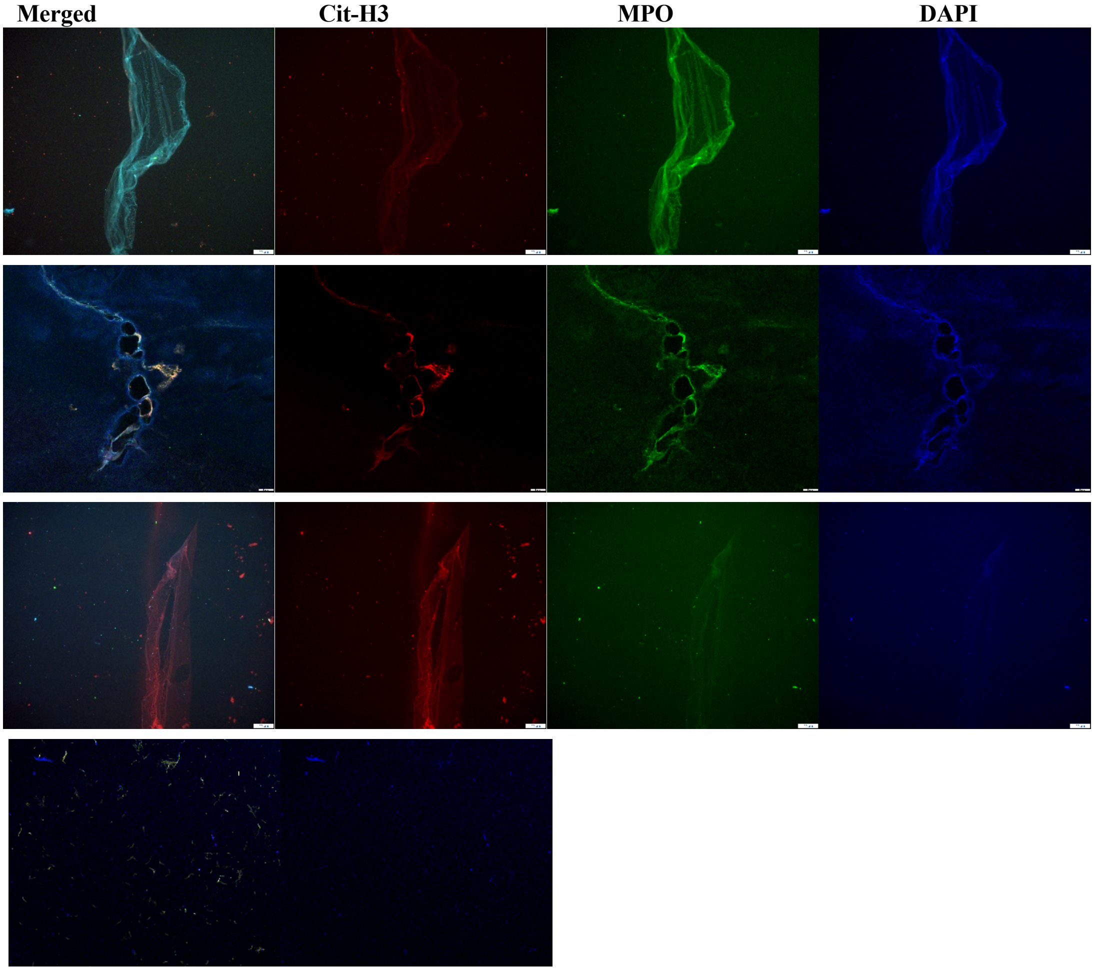

III.3 -/-

III.6 -/-

III.7 -/-

II M +/-

Family B

Merged **Cit-H3** **MPO** **DAPI**

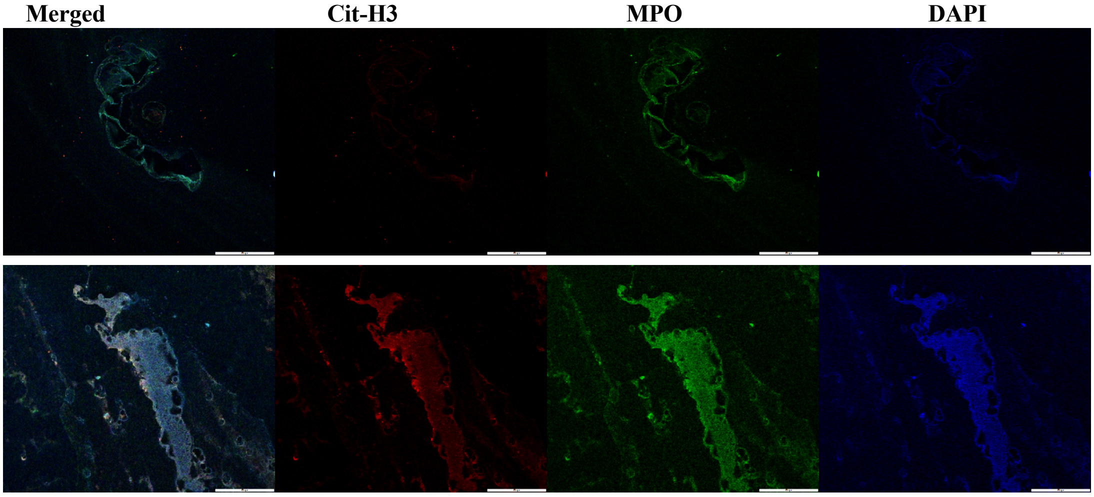

III.4 -/-

III.5 -/-

III.2 -/-

II M +/-

Control

+/+

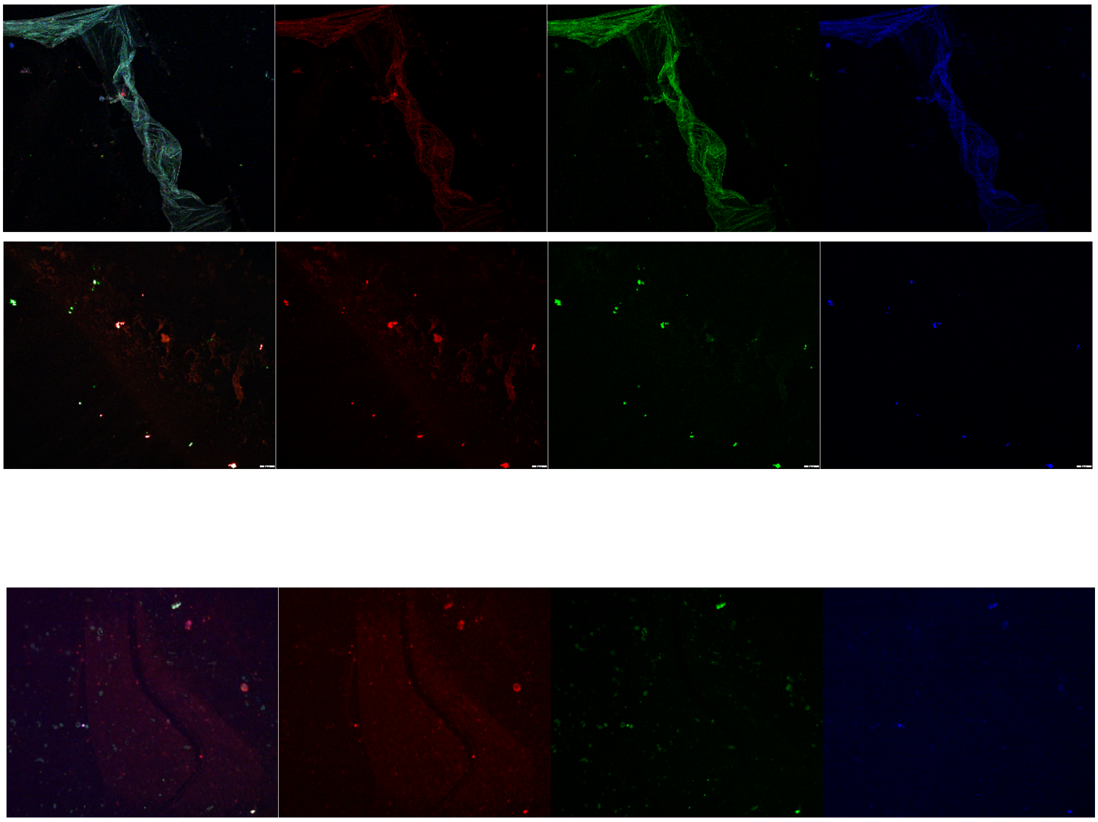

Supplement: online supplemental file 1 [file lupus-12-1-s001.pdf]
